# The role of plasma inflammatory markers in late-life depression and progression to dementia: a 3-year follow-up study

**DOI:** 10.1192/j.eurpsy.2025.531

**Published:** 2025-08-26

**Authors:** M. Bocharova, D. Aarsland, A. Young, J. Hodsoll, K. Engedal, J. T. O’Brien, G. Selbæk, A.-V. Idland, L. O. Watne, T. Borza

**Affiliations:** 1 Centre for Healthy Brain Ageing; 2 Centre for Affective Disorders; 3 Biostatistics & Health Informatics, King’s College London, London, United Kingdom; 4 Norwegian National Advisory Unit on Ageing and Health, Vestfold Hospital Trust, Tønsberg, Norway; 5 Department of Psychiatry- School of Clinical Medicine, University of Cambridge, Cambridge, United Kingdom; 6 Department of Geriatric Medicine, Oslo University Hospital, Oslo, Norway; 7 Department of Geriatric Medicine, Oslo University Hospital, Oslo, United Kingdom; 8 Institute of Clinical Medicine, Campus Ahus, University of Oslo, Oslo; 9 Research Centre for Age-related Functional Decline and Disease, Innlandet Hospital Trust- Sanderud, Ottestad, Norway

## Abstract

**Introduction:**

Late-life depression (LLD) has been linked to increased likelihood of dementia, although mechanisms responsible for this association remain largely unknown. One feature frequently observed in both LLD and dementia is elevated levels of plasma inflammatory markers.

**Objectives:**

The present study aimed to compare the levels of 12 plasma inflammatory markers between older people with LLD and controls, and to explore whether these markers, along with clinical characteristics, can predict dementia in patients with LLD within 3 years of follow-up.

**Methods:**

Using multiple linear regression with stepwise adjustment (for age, gender, smoking status, and physical comorbidities), we compared levels of plasma inflammatory markers (IL-1β, IL-1ra, IL-6, IL-10, IL-17a, IL-18, IL-33, TNFα, CD40L, IFN-γ, CCL-2 and CCL-4) between 136 older inpatients admitted to psychiatric units for LLD (PRODE cohort) and 103 cognitively healthy non-depressed controls (COGNORM cohort). In the PRODE cohort, follow-up data was available for 139 patients (of them 123 had data on baseline plasma inflammatory markers); 36 (25.9%) developed dementia by year 3. Using Cox proportional hazards regression, we explored whether inflammatory markers and clinical characteristics of LLD (age of onset, treatment response, number of episodes) predicted progression to dementia during follow-up.

**Results:**

Levels of IL-1ra, CCL-2, CCL-4, IFN-γ and IL-17a were significantly higher in LLD patients compared to controls in all models (See Fig 1); IL-33 was significantly elevated in most models. None of the baseline plasma inflammatory markers predicted progression from LLD to dementia. Among clinical features, only improvement in MADRS score at discharge (HR = 0.95, 95% CI 0.91-0.99) and treatment response (HR 0.45, 95%CI 0.21 – 0.98) were associated with lower chance of progression to dementia in fully adjusted models; age of onset and number of previous episodes were not significant predictors of dementia at follow-up.

**Image 1:**

**Figure:**
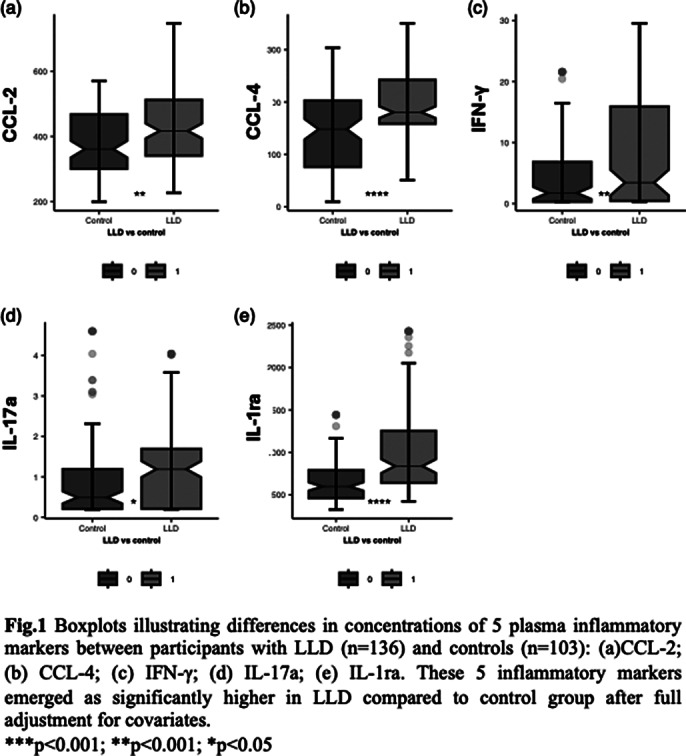

**Conclusions:**

This study demonstrated an increase in plasma inflammatory markers in LLD but did not find evidence they could predict dementia at follow-up.

**Disclosure of Interest:**

None Declared

